# Paradigm shifts in pathophysiology and management of atrial fibrillation—a tale of the RACE trials in the Netherlands

**DOI:** 10.1007/s12471-020-01476-0

**Published:** 2020-08-11

**Authors:** H. J. G. M. Crijns, I. C. Van Gelder

**Affiliations:** 1 Department of Cardiology and the Cardiovascular Research Institute Maastricht, Maastricht University Medical Center, Maastricht, The Netherlands; 2 Department of Cardiology, Thoraxcenter, University of Groningen, University Medical Center Groningen, Groningen, The Netherlands

**Keywords:** Atrial fibrillation, Rate control, Rhythm control, Heart failure, Quality of life, Randomised clinical trial

## Abstract

In the past 20 years the Netherlands-based RACE trials have investigated important concepts in clinical atrial fibrillation (AF). Their scope ranged from rhythm versus rate control to early or delayed cardioversion and also included early comprehensive management of AF in two trials, one focusing on early ‘upstream therapy’ and risk factor management and the other on integrated chronic nurse-led care. Studies were mostly triggered by simple clinical observations including futility of electrical cardioversion in persistent AF; many patients with permanent AF tolerating day-after-day ‘uncontrolled’ resting heart rates of up till 110 beats/min; patients being threatened more by vascular risks than AF itself; and insufficient guideline-based treatments for AF. Also the observation that recent-onset atrial fibrillation generally converts spontaneously, obviating cardioversion, triggered one of the studies. The RACE trials shifted a number of paradigms and by that could change the AF guidelines. The initial ‘shock-and-forget’ attitude made place for increased attention for anticoagulation, and in turn, broader vascular risks were recognised. In a nutshell, the adage eventually became: ‘look beyond the ECG, treat the patient’.

**Electronic supplementary material:**

The online version of this article (10.1007/s12471-020-01476-0) contains supplementary material, which is available to authorized users.

## Dutch contribution to the field

- The revolutionary notion of ‘electrical remodelling’ inspired many investigators to develop new clinical concepts including ‘the second factor’ to complement electrical remodelling, AF progression as an endpoint in clinical trials, and early AF management and early comprehensive upstream therapy to improve prognosis.
- Several significant paradigm shifts in AF treatment happened: rhythm control was offset by rate control in persistent AF, aggressive by lenient rate control in permanent AF, and acute restoration of sinus rhythm by the wait-and-see approach in recent-onset AF. Lately the concept that electrical cardioversion should be considered a diagnostic rather than a therapeutic procedure emerged. The RACE studies also fed the notion that besides stroke, AF patients are even more threatened by heart failure and cardiovascular death.
- Cardiovascular risk scores to steer AF management were developed and swept the world, among which CHA_2_DS_2_-VASc, HAS-BLED and HATCH scores.
- The RACE consortium strongly advocated nurse-led integrated chronic care for atrial fibrillation and demonstrated its overall effectiveness; nurses steering integrated care perform better than stand-alone doctors.

## Introduction

The first RACE study [1] originated from the Academic Hospital Groningen and spread rapidly across the Netherlands. All studies from the RACE consortium were strongly supported by the Working Group Cardiology Centres in the Netherlands (WCN), several non-WCN cardiology centres as well as the academic centres in Maastricht, both centres in Amsterdam, and Nijmegen. Per RACE study the participation of centres varied but the group remained cohesive. The Netherlands Heart Institute (the former Interuniversity Cardiology Institute in the Netherlands—ICIN) formed the scientific basis and a meeting place for all investigators and research nurses. The initial acronym RACE stands for ‘RAte Control versus Electrical cardioversion for persistent atrial fibrillation’. We kept the acronym for the subsequent studies since the consortium partners and theme of study remained connected and constant through time. Most RACE trials were advised by a data safety monitoring committee of which Hein Wellens—now deceased—was a prominent member. Many medical scientists served on the respective endpoint event committees (Electronic Supplementary Material). A very important scientist for all RACE studies was Jan Tijssen, at present emeritus professor at the University of Amsterdam. His cardiovascular biostatistical insights and excellent knowledge of the field yielded robust biostatistics and by that a solid basis for high ranking publications of the results.

The RACE trials investigated and challenged important concepts in clinical atrial fibrillation (Tab. 1). In the early years of our careers, cardiologists looked upon atrial fibrillation (AF) as an arrhythmia for which rhythm control, especially antiarrhythmic drugs and electrical cardioversion (ECV), were most important (‘shock-and-forget’). Subsequently, the focus shifted massively towards anticoagulation, fuelled by observations that patients had spontaneous or cardioversion-provoked cardioembolic strokes. The initial ‘*big 6’* stroke trials [2] were—much later—followed by industry-academy alliances propelling the trials on non-vitamin K dependent anticoagulants. Thereafter, driven by observations from the EuroHeartSurvey on AF [3–5] as well as the two initial RACE studies [1, 6], we began to focus on comprehensive management of AF, i.e. treatments in addition to rate and rhythm control, and anticoagulation (‘look beyond the ECG, treat the patient’) [7]. For us a very important trigger was the observation that rates of mortality and heart failure were significantly higher [1, 4, 6] than the rate of stroke, and that patients suffer stroke despite anticoagulation. Then, comprehensive risk factor management—including rehabilitation and lifestyle—was studied in RACE3 [7, 8], in parallel with the study of integrated chronic care for AF (RACE4) [9]. RACE V, still ongoing, concerns a registry with the concept of hypercoagulation as a central mechanism for atrial remodelling and AF progression. RACE6-CV@H (cardioversion at home) is a proof-of-concept study into ECV performed at patients’ homes for their convenience and to test reallocation of hospital care. Conceived well before the 2020 COVID-19 pandemic, its concept perfectly fits the current hospital-at-home care strategies. Also in the early versus delayed cardioversion trial (RACE7-ACWAS) [10, 11] avoidable hospital procedures were the focus. As we became senior scientists, our successors Kevin Vernooy, Michiel Rienstra and Dominik Linz recently started two RACE studies which focus on ablation in heart failure (RACE8) and tele-checked rate control of recent-onset AF (RACE9). The present paper gives a perspective of all RACE studies and their contribution to the field.

**Table 1:** Concepts challenged and hypotheses tested by the RACE trials, with results or subsequent changes

	Concept/Hypothesis	Result/Change	References
*RACE*	Sinus rhythm better than AF	Rate control not inferior to rhythm control	[1, 21]
	Mending the rhythm improves prognosis	No change	[18]
	Rhythm control affects sudden death?	No impact	[42]
	Sex differences may exist in rate and rhythm control outcomes	Females suffer excess cardiovascular events under rhythm control	[26]
	Rhythm control gives better QoL	No difference with RC	[25]
	Costs lower with RC	Costs proven lower with RC	[24]
	RC may be deleterious in patients with CHF	In patients with mild to moderate CHF, RC is not inferior to rhythm control	[43]
	Clinical lone AF is not associated with cardiovascular events	Clinical lone AF is associated with bleeding and thromboembolism	[44]
	Underlying comorbidities may affect outcome differently between rate and rhythm control	In hypertensives, pharmacological rhythm control is associated with cardiovascular morbidity/mortality; consider default RC	[45]
	Anticoagulation should be bridged around surgery	Extremely low perioperative thromboembolism risk; interruption of warfarin less dangerous than previously thought	[22]
	Strict RC is standard of care (comparison of RC in RACE (lenient) and AFFIRM (strict))	Strict RC causes CV events, including excess artificial pacemaker implantations	[28]
*RACE-II*	Strict rate control with resting heart rate in AF recommended as <80 bpm	Lenient RC not inferior to strict RC	[6, 46, 47]
	Strict RC in AF and HF improves symptoms, CV prognosis and QoL	No beneficial effect of strict RC in permanent AF patients	[48]
	Strict RC improves QoL	Stringency of RC does not affect QoL; symptoms, sex, age, underlying disease affect QoL	[32]
	Strict RC may fail, which predisposes to events	Strict RC fails in 33% of patients but is not associated with events; lenient RC is preferred	[30]
	Digoxin affects morbidity and mortality	The use of digoxin was not associated with increased morbidity and mortality	[31]
*RACE3*	Targeted ‘upstream therapy’ for secondary AF prevention unproven	First study to show improved rhythm outcome with upstream therapy	[7, 8, 49]
	Optimal upstream therapy may not be feasible in all patients	Upstream therapy feasible in 57% of patients; it is associated with enhanced rhythm outcome	[37]
	QoL change uncertain	Targeted therapy improves QoL, not necessarily through obtaining sinus rhythm	[33, 38]
*RACE4*	Doctors manage AF better than nurses	(Experienced) nurses manage better	[9, 39, 41]
*RACE‑V*	AF progression is driven by hypercoagulation	Expected change: anticoagulation prevents AF progressions, not only stroke	
	Uncertain role for ILR	Expected: ILR detects temporal types of AF	
*RACE6-* *CV@H*	Electrical cardioversion must be done in-hospital	Expected change: cardioversion can be safely performed at home	
*RACE7-* *ACWAS*	Early cardioversion better than delayed cardioversion for recent-onset AF	Delayed cardioversion not inferior to early cardioversion	[10, 11, 50]
*RACE8-* *HF*	Cryoballoon PVI improves prognosis in persistent AF and heart failure	Expected change: uncertain, remains to be seen	
*RACE9*	Cardioversion (early or delayed) remains a key procedure in recent-onset AF	Expected change: interventional rhythm control has no significant role in stable recent-onset AF	
	Telemonitoring in management of recent-onset AF has—as yet—no place!	Expected change: telemonitoring prevents needless interventions and keeps patients safely out-of-hospital	

*AF* atrial fibrillation, *bpm* beats per minute, *CHF* congestive heart failure, *ILR* implantable loop recorder, *LV* left ventricle, *PVI* pulmonary vein isolation, ablation therapy, *QoL* quality of life, *RC* rate control

## The first RACE study: setting the stage, execution and aftermath

**Fig. 1:**
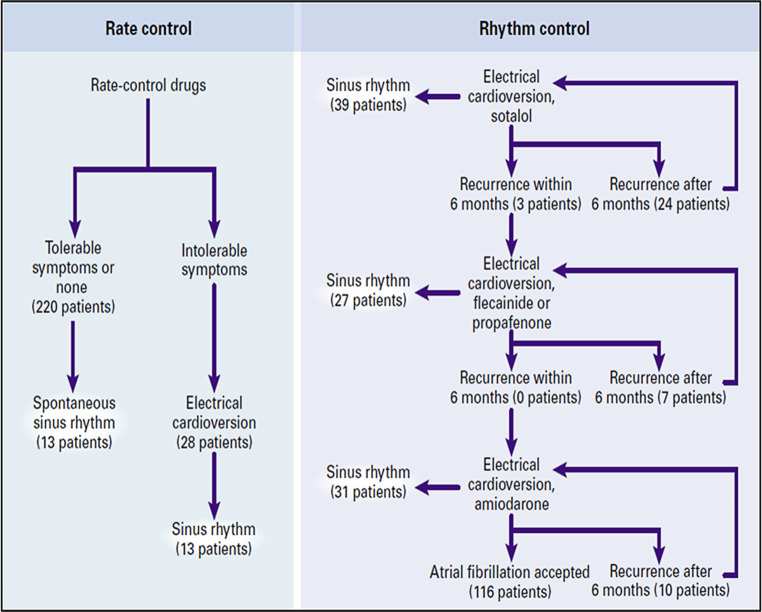
Rate and rhythm control management in RACE [1]. In the rate control strategy patients with intolerable symptoms were allowed to undergo cardioversion. This was not considered a cross-over but an essential part of that strategy. The rhythm control strategy consisted of serial cardioversion supported by antiarrhythmic drugs. Drugs changed only if recurrence happened under an adequate dosage and only in case of an early recurrence, defined as happening within 6 months. Cardioversion patients resorting to rate control (‘atrial fibrillation accepted’) were not considered cross-overs since that was an essential part of the rhythm control strategy, paralleling clinical practice. Reprinted with permission of the Massachusetts Medical Society

**Fig. 2:**
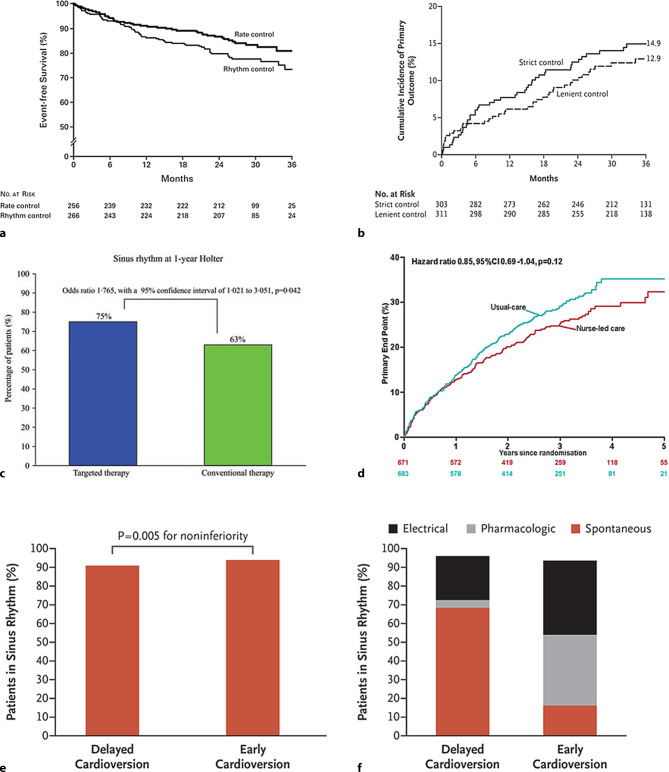
Main results from 5 RACE trials. **a** RACE: rate control is non-inferior to rhythm control [1]; **b** RACE-II: lenient rate control is non-inferior to strict rate control [6]; **c** RACE3: upstream therapy was associated with a modest improvement of rhythm control [8]; **d** RACE4: nurse-led care appeared not superior to usual-care provided by a cardiologist, although in proficient centres, nurse-led care was associated with significantly fewer events [9]; **e** RACE7-ACWAS: sinus rhythm at 4 weeks after delayed cardioversion and standard-of-care early cardioversion did not differ; **f** RACE7-ACWAS: sinus rhythm during index visit, according to type of cardioversion – in 69% of patients in the delayed cardioversion strategy an intervention may be avoided. In contrast, an early intervention allows only for 16% spontaneous conversion with 80% obligatory cardioversions [10]. Reprinted with permission of the Massachusetts Medical Society (RACE, RACE-II and RACE7-ACWAS) and Oxford University Press (RACE3 and RACE4)

The first RACE trial, evaluating whether simple rate control was non-inferior to complex rhythm control, was stimulated by several clinical observations. First and foremost, long-term maintenance of sinus rhythm after ECV was disappointingly low, with 90% of patients having a recurrence of persistent AF after 4 years [12]. With that, cost-effectiveness as well as the effect on quality of life of cardioversion were questioned. In fact it was Henk Lie, former head of the Department of Cardiology in Groningen and our well-respected PhD and cardiology residency supervisor, who challenged us by asking the simple question ‘Cardioversion …, does it help?’ During the preparation of the RACE trial, friend and colleague scientist Maurits Allessie, Department of Physiology in Maastricht, cautioned us that the trial might come too early since the therapies to maintain sinus rhythm were still unsatisfactory. At that time, the AFFIRM trial [13] was about to start and therefore we were eager to go through with RACE. On the basis of Allessie’s advice we optimised the rhythm control treatment requirements for the investigators. ECV was performed in a serial manner with repeat cardioversion as needed and a change of prophylactic antiarrhythmic if the recurrence was early, i.e. within 6 months (Fig. 1; [1, 12, 14]). If, on the other hand, a recurrence happened beyond 6 months of sinus rhythm, the drug in use was left unchanged. We asked investigators to maximise efforts to keep patients in sinus rhythm by performing repeat cardioversion as quickly as possible after a recurrence and tailor antiarrhythmic drug treatment to the patient, avoiding both underdosing as well as side effects. During those years catheter ablation was not widely used for AF. Rate control was performed with a lenient target of 100 beats per minute (bpm). A non-electrical primary endpoint was chosen in RACE since it is not the eventual rhythm during follow-up that counts but rather longevity of the patient and avoiding severe adverse events including heart failure, stroke, bleeding, side effects of all AF drugs as well as pacemaker implantation. Back then, using cardiovascular hospitalisation as an endpoint in arrhythmia trials came into consideration [15]. For many arrhythmologists that may have felt counter-intuitive (a non-electrical endpoint after an electrical intervention) but it was clinically most sensible for evaluating interventions in arrhythmia trials. As expected, rhythm control was associated with significantly more patients in sinus rhythm compared with rate control (39% versus 10%) [1]. However, there was no difference in the composite endpoint cardiovascular death and hospitalisation (Fig. 2). Critics of the RACE and AFFIRM [16] trials reasoned that the neutral results were due to the low cumulative time patients spent in sinus rhythm during follow-up. Post-hoc analysis of AFFIRM showed an association between sinus rhythm and survival [17]. In contrast, ‘mending the rhythm’ was not associated with event-free survival in RACE [18]. What critics did not understand was that staying in sinus rhythm is not only by virtue of the intervention but is heavily influenced by the underlying cardiovascular condition at the outset of the procedure, in particular the state of atrial remodelling. They also misunderstood the fact that RACE compared two strategies rather than the very acts of rate control (should yield appropriate rate) and rhythm control (should give permanent sinus rhythm). Like AFFIRM (and many later trials in this area, including CASTLE-AF) [19], RACE was a *strategy evaluation* and not an evaluation of the efficacy of cardioversion in maintaining sinus rhythm or of rate control to obtain an acceptable heart frequency. Also it was not an exercise in keeping the rate control arm patients away from cardioversion if that was deemed clinically indicated nor an exercise in obstructing channelling a rhythm control patient to rate control after repeated failure of cardioversion. In this respect, concepts like ‘per protocol analysis’ and ‘cross-over’ are pointless since failing rate or rhythm control may all be the outcome of an otherwise perfectly executed strategy. Post-hoc per-protocol analyses as performed in CABANA [20] are misleading and unfortunately they feed endless and perfectly fruitless debates. The bottom-line here is that sinus rhythm is frequently a marker of survival, and not the mechanism of survival!

Numerous sub-analyses were performed in the RACE population (Tab. 1). One of the main lessons learned from RACE is that anticoagulation must be continued if stroke risk factors are present even if patients maintain sinus rhythm [21]. RACE provided an excellent opportunity to check thromboembolism in almost 94 patients undergoing 121 non-cardiac surgeries. It was found that perioperative interruption of anticoagulation is far less dangerous than previously believed [22]. The latter findings were in line with the subsequent study by Douketis et al. [23] which led to widespread abandoning bridging of anticoagulation. A cost analysis indicated that costs were lower in the rate control arm compared with the rhythm control arm [24]. Over 2.3 years of follow-up, the mean costs per patient were € 7386 under rate control and € 8284 under rhythm control. Under rhythm control, more costs were generated due to electrical cardioversions, hospital admissions and antiarrhythmic medication. Quality of life appeared unaffected by strategy [25]. A sex-related sub-study showed that female patients with persistent AF had significantly higher cardiovascular morbidity and mortality under rhythm control compared with rate control [26]. Events were mainly heart failure, thromboembolism and adverse effects of antiarrhythmic drugs. Presumed mechanisms include higher prevalence of diastolic heart failure not amenable to rhythm control and heart failure associated with AF recurrence, lower rate of adequate anticoagulation in females, bradycardias and higher pacemaker implantation rates due to unmasking of chronotropic and dromotropic incompetence, mainly in females. Taken together, rhythm control should be applied judiciously in females, and when in doubt be avoided.

## The birth of RACE-II

RACE-II was ushered in by a criticism on RACE indicating the rate control arm was too lenient allowing an upper limit for resting rate in AF of 100 beats per minute (bpm). The AF guidelines at that time recommended an upper limit of 80 bpm [27]. Subsequently we constructed a lenient rate control arm in RACE-II with a limit at 110 bpm. The latter was based on the observation that most of our patients did well using a target resting heart rate below 100–110 bpm. We also reasoned that an at-all-costs strict rate control would be associated with iatrogenic bradycardia and excess pacemaker implants. A post-hoc comparison of the rate control arms of RACE (lenient control, below 100 bpm) and AFFIRM (strict rate control, resting heart rate below 80 bpm) showed a significantly lower heart rate but a higher composite of cardiovascular death, hospitalisation and myocardial infarction in AFFIRM compared with RACE (34 vs 25%) and conspicuously more patients in need of pacemaker therapy (11 vs 1% over 3 years, *p* < 0.009) [28]. A few years later, in a randomised controlled comparison, RACE-II showed that lenient rate control is non-inferior to strict rate control (Fig. 2; [6]), which led to a change in the AF guidelines [29]. RACE-II has remained unique: unfortunately no further randomised clinical trials have been performed in this area. Note that patients with severe heart failure were not included.

Sub-analyses of RACE-II are shown in Tab. 1. An interesting finding was that strict rate control fails in up to one third of patients, i.e. a resting heart rate below 80 bpm could not be achieved. In line with the main results of RACE-II, failure of strict rate control was not associated with excess events compared with successful strict or lenient rate control [30]. Another interesting finding is that in stable permanent AF digoxin may be used safely to control heart rate [31]. This is in contrast to several post-hoc analyses from large studies suggesting that digoxin increases mortality, but all of these studies on AF and digoxin are post-hoc and many suffer from extensive selection biases or bias by indication. Note that both in RACE and RACE-II there was no difference in quality of life between the intervention and control groups [25, 32]. In contrast, RACE3 (below) showed that quality of life improved under upstream therapy compared with control, an effect which was independent of whether patients maintained sinus rhythm during follow-up or not [33].

## Upstream to RACE3

**Fig. 3:**
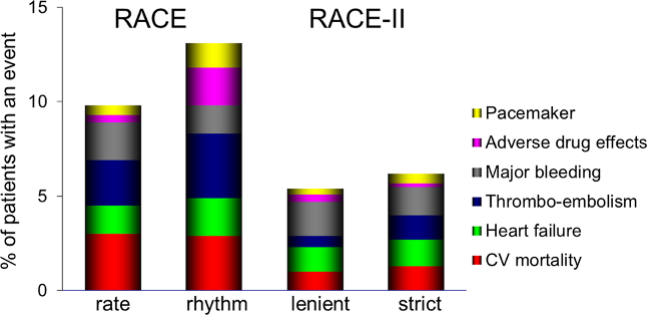
Over 2.3 years follow-up, RACE (published 2002) [1] had significantly more endpoint events than RACE-II (published 2010) [6], despite baseline cardiovascular risk being comparable and with an even higher intrinsic risk in permanent AF in RACE-II. This time-dependent change was likely due to the more widespread use of anticoagulants in RACE-II with far fewer thromboembolic complications but coming at the cost of (unchanged) bleeding. In addition, the more extensive prescription of renin-angiotensin system blockers in RACE-II also contributed. RACE and RACE-II included 522 and 614 patients, average age 68 and 68 years, average atrial fibrillation duration 11 and 18 months, CHA_2_DS_2_-VASc 1.4 and 1.4, all respectively. Overall anticoagulant use varied in RACE between 96–99% in rate control and 86–99% in rhythm control and in RACE-II it was constant at 99% of time

An unpublished comparison of RACE and RACE-II (Fig. 3) showed that over the 10 years between those two studies sustained rather than interrupted anticoagulation as well as management of high blood pressure and heart failure with renin-angiotensin system inhibition had improved prognosis: less stroke and bleeding, fewer admissions for myocardial infarction or heart failure, and fewer severe side effects of drugs. The scientific community started to see AF as a vascular disease rather than an arrhythmia. The latter held for the majority of patients while, in only relatively few patients, AF is an exclusively electrical disease in which the electrical abnormality precedes onset of AF. In vascular AF, however, the underlying heart disease precedes AF by many years, usually more than a decade or two, and that mostly concerns hypertension, atherosclerotic heart disease, obesity or heart failure. The term vascular AF refers not only to underlying vascular disease but also to the notion that vascular remodelling (in particular left atrial dilation and fibrosis) precedes the onset of AF by decades, setting the electrophysiological stage for AF. Cosio and Crijns, supported by a group of experts in the field, were among the first to recognise that in clinical practice vascular disease precedes AF in many instances (their Fig. 3; [34]). Once AF emerges, most patients already suffer from vascular remodelling. These notions were fed by remarkable findings from studies such as the LIFE trial [35] indicating that at similar blood pressure reduction in hypertensive patients also suffering from left ventricular hypertrophy, the angiotensin receptor blocker (ARB) losartan halved the incidence of AF compared with the beta-blocker atenolol (primary prevention). Of even greater significance, those investigators showed that in patients with incident AF during the study, strokes were halved by losartan compared with atenolol, strongly suggesting that ARBs, as non-antithrombotic drugs, may help to prevent stroke through their vascular protective effects. In 2007, Savelieva and Camm introduced the notion of ‘upstream therapy’, indicating that through primary prevention, AF and its cardiovascular sequels can be effectively reduced. Typically, upstream therapy and risk factor management would target remodelling processes through reduction of inflammation, oxidative stress and extracellular matrix remodelling driven atrial fibrosis, using ARBs, mineralocorticoid receptor antagonists (MRAs) and statins [36]. In addition, cardiovascular risk factors become reduced. In RACE3 we reasoned that single-element upstream therapy would leave other remodelling pathways and other risk factors open [7]. Therefore, we hypothesised that a combination of different classes of upstream therapies would have synergistic effects on the atrial substrate and thereby decrease AF. In addition to anti-remodelling drugs and optimised risk factor management, we introduced a lifestyle intervention with cardiac rehabilitation since regular exercise may reduce AF. We tested our hypothesis in patients with early persistent AF and early heart failure (predominantly heart failure with preserved ejection fraction, HFpEF) since in advanced stages of these diseases AF would no longer be amenable to upstream therapy. Note that the primary aim was to reduce recurrent AF after cardioversion. RACE3 showed that upstream therapy significantly reduces risk factors (blood pressure, cholesterol) as well as recurrent AF (Fig. 2). Therefore, lifestyle intervention, ARBs, MRAs and statins all should be considered in persistent AF and stable heart failure. Obviously, this composite of upstream therapies yielded only a moderate rhythm control effect and cannot replace antiarrhythmic drug therapy and catheter ablation. It, however, fits perfectly into a comprehensive therapeutic strategy of treating not only AF but also underlying comorbidities and risk factors. It needs to be seen whether better maintenance of sinus rhythm using upstream therapy will result in improved survival. This holds especially since a post-hoc sub-analysis showed that only 57% of all patients in the interventional group reached their upstream targets (i.e. had optimal therapy) [37]. Another sub-analysis [33, 38] showed that quality of life improves significantly with the multi-faceted upstream therapy compared with control treatment. This was seen independent from rhythm outcome, meaning that other pathways than rhythm control seem active in maintaining quality of life under targeted upstream therapy (Tab. 1).

## The Hendriks study and RACE4

In 2012—under the supervision of Robert Tieleman—Jeroen Hendriks reported that guideline-based, ICT-supported, physician-supervised, nurse-driven care for AF was superior to usual-care provided by a cardiologist [39]. That then unique study was criticised for being mono-centre. In RACE4 we adopted the approach of integrated chronic care in a multi-centre trial. RACE4 showed that among patients recently referred for management of first-detected AF, nurse-led care did not significantly reduce cardiovascular death or hospitalisation compared with usual-care (Fig. 2). Remarkably, nurse-led care did not enhance patient knowledge on AF or quality of life. Nevertheless, a predefined exploratory analysis showed that centres with higher proficiency and experience in nurse-led care performed significantly better concerning cardiovascular endpoints than less experienced centres. On the basis of the similar event rates between both approaches, one may conclude that nurse-led care is a safe and effective way of providing care for patients with AF. Therefore, continued education and sharing of knowledge between centres are key to increasing the impact of nurse-led integrated care in AF clinics [40]. Patient numbers are growing and the average age of AF patients is increasing. Therefore, nurse-led integrated chronic care will become inevitable to enable cost-effective and widely accessible care for AF in the future [41].

## RACE7-ACWAS—to cardiovert now or later?

Patients reporting to the emergency department (ED) frequently convert spontaneously under the eyes of the attending physician. Also, in the outpatient setting, many patients report self-termination of AF for which they do not even bother to come to the hospital. Obviously, mainly patients with prominent symptoms present to the ED and cardioversion is performed almost automatically by a willing team eager to clear the department. However, the opportunity to observe spontaneous conversion is wasted, which in itself may reveal important information for chronic management. Acute conversion also distracts from what really matters: the need for anticoagulation and how to manage recurrent episodes. These two approaches were evaluated in the RACE7-ACWAS trial [11]. Fig. 2 shows the main result. Wait-and-see with delayed cardioversion as needed appeared non-inferior to early or acute cardioversion in terms of sinus rhythm at one month after the index visit to the ED. The study provided insight into the advantages and disadvantages of both approaches (Tab. 2). One important aspect is that patient and physician can take an informed shared decision on the management of preference in the ED. In addition, patients having experienced a spontaneous conversion will more likely stay at home in case of a new episode. All medical information on episodes emerging over time may feed into a decision for interventional therapy or not. An ongoing cost-effectiveness analysis will provide insight into reduction of costs of the wait-and-see strategy.

**Table 2:** Comparison of advantages and disadvantages of early and delayed conversion (after RACE7-ACWAS)

Early cardioversion	Delayed cardioversion
Foreshortens time to conversion	Number of patients eventually in SR not affected
Earlier elimination of fibrillation complaints?	Complaints equally reduced by reassurance and rate control
Prevention of tachycardia-related adverse events?	Adverse events low and similar with both approaches
Prompter discharge from the ED?	Even prompter discharge in delayed group
Total time spent in ED shorter? No 2nd day needed	Total time spent shorter with delayed strategy, including 2nd day
Prevents AF progression?	No persistent AF observed during FU
Shorter time in AF prevents stroke?	Appropriate OAC prevents stroke; besides, AF duration is not a determinant of stroke
Rate control? Mostly not looked after although 1/3 of AF recurs <30 days!	Rate control prevents high rates during recurrence
Quality of life better?	Quality of life not different
Burden to ED for more frequent cardioversions	Early discharge and planned CV reduces burden for ED
Associated with failure to initiate anticoagulation	Idem (a bit less undertreatment in 1st detected AF)

*AF* atrial fibrillation, *CV* cardioversion,* ED* emergency department, *OAC* oral anticoagulation, *SR* sinus rhythm

## The RACE trials—a clinical perspective of cardioversion

Rhythm control by ECV is still seen as a therapeutic procedure although several RACE trials have shown that it is often therapeutically futile. Nevertheless, ECV may have an important application as a diagnostic procedure. Firstly, it may help to establish whether the arrhythmia causes symptoms, e.g. by assessing the symptom/rhythm correlation in the work-up towards an ablation. In persistent AF it is frequently difficult to establish whether eliminating AF (e.g. by ablation with its intrinsic risks) will reduce symptoms since patients’ complaints may be related to other mechanisms, conspicuously to concomitant HFpEF. HFpEF is very frequently associated with AF. Secondly, a diagnostic ECV may underpin the diagnosis of tachycardiomyopathy due to AF in patients suffering from AF and heart failure with a reduced left ventricular ejection fraction (HFrEF). If, after ECV, the left ventricular ejection fraction improves, tachycardiomyopathy with HFrEF is a most probable diagnosis. To prevent future recurrences of tachycardiomyopathy, ablation therapy may then be warranted or at least stringent rate control is needed if ablation fails or is not considered. Although this all sounds great, its wider application needs a change of attitude among attending physicians. Up till now, the greatest challenge is to bridge the disconnect between the world of arrhythmologists and heart failure cardiologists, with only very few heart failure specialists offering their HFrEF or HFpEF patients also suffering from AF a way out of their electrical heart failure. Vice versa, electrophysiologists should look beyond the ECG and provide heart failure and other risk mitigating treatments more attentively.

## Conclusion

The Netherlands RACE trials were a concerted action of many centres in the Netherlands and challenged established clinical concepts. Starting from simple clinical observations with the perspective of improving care, robust clinical trials could be constructed. As they moved forward they helped to change guidelines and improve care for AF patients.

## Caption Electronic Supplementary Material

The electronic supplement provides the overisight of all RACE trials, including information on participating centres, contributing scientists, committee members and financial support.
