# Supplementary material for: Paradigm shifts in pathophysiology and management of atrial fibrillation—a tale of the RACE trials in the Netherlands
Source: Neth Heart J. 2020 Aug 11;28(Suppl 1):3–12. doi: 10.1007/s12471-020-01476-0 (PMC6940410; doi:10.1007/s12471-020-01476-0)
Supplement: Supplementary file 1 — The electronic supplement provides the overisight of all RACE trials, including information on participating centres, contributing scientists, committee members and financial support. [file 12471_2020_1476_MOESM1_ESM.docx]

Appendix

Sponsors of the RACE trials

RACE I: Supported by grants from the Center for Health Care Insurance (OG96-047) and the Interuniversity Cardiology Institute, the Netherlands, and by an unrestricted grant from 3M Pharma, the Netherlands.

RACE II: Supported by the Netherlands Heart Foundation (2003B118) and unrestricted educational grants from AstraZeneca, Biotronik,

Boehringer Ingelheim, Boston Scientific, Medtronic, Roche, and Sanofi Aventis France (paid to the Interuniversity Cardiology Institute of the Netherlands).

RACE III: The Netherlands Heart Foundation (Grant 2008B035); the Netherlands Heart Institute; Unrestricted grants from AstraZeneca, Bayer, Biotronik, Boehringer-Ingelheim, Boston Scientific, Medtronic, Sanofi-Aventis, St Jude Medical paid to the Netherlands Heart Institute.

RACE 4: This research was supported by Netherlands healthcare insurance companies (DSW, ACHMEA, and CZ), Boehringer Ingelheim, Bayer, Pfizer, Bristol-Myers Squibb, and Daiichi-Sankyo.

RACE V: We acknowledge the support from the Netherlands Cardiovascular Research Initiative: an initiative with support of the Dutch Heart Foundation, CVON 2014-9: Reappraisal of Atrial Fibrillation: interaction between hyperCoagulability, Electrical remodelling, and Vascular destabilisation in the progression of AF (RACE V). Unrestricted grant support from Medtronic Trading NL B.V.

RACE 6 CV@H is supported by the Ambulancedienst Oost, Hengelo.

RACE 7: Supported by a grant (837002524) from the Netherlands Organization for Health Research and Development–Health Care Efficiency Research Program and by the Maastricht University Medical Center. Boehringer Ingelheim provided some of the remote

monitoring devices.

Participants and committee members per RACE trial

**RACE**

The following persons participated in the Rate Control versus Electrical

Cardioversion for Persistent Atrial Fibrillation Study (the numbers in parentheses

indicate the numbers of patients enrolled): *University Hospital, Groningen*

— H. Crijns, I. Van Gelder, V. Hagens, T. Kingma (74); *St. Antonius*

*Hospital, Nieuwegein* — J. Lindeboom, J. Kingma (36); *Hospital*

*Midden-Twente, Hengelo* — S. Said (34); *Rijnstate Hospital, Arnhem* — H.

Bosker (31); *Medisch Spectrum Twente Hospital, Enschede* — A. Timmermans

(31); *Twenteborg Hospital, Almelo* — J. Darmanata, G. Linssen, B. de Rode

(30); *Ignatius Hospital, Breda* — R. Wielinga (24); *Isala Hospital, Zwolle*

— A. van ’t Hof, M. Vet (24); *Oosterschelde Hospital, Goes* — E. Bruyns,

A. Liem (22); *Free University Medical Center, Amsterdam* — M. Mihciokur,

O. Kamp (21); *Stichting Deventer Hospitals, Deventer* — E. Badings, D. Lok

(20); *Canisius Wilhelmina Hospital, Nijmegen* — D. Hertzberger (19); *St. Lucas*

*Hospital, Winschoten* — T. Bouwmeester, A. van der Galiën (18); *Catharina*

*Hospital, Eindhoven* — A. Meyer, F. Bracke (11); *Scheper Hospital,*

*Emmen —* M. Nagelsmit (11); *Onze Lieve Vrouwe Hospital, Amsterdam* —

T. Slagboom (9); *Hospital Hilversum, Hilversum* — K. Liem (9); *Antonius*

*Hospital, Sneek* — B. Cernohorsky (9); *Reinier de Graaf Hospital, Delft* —

D. Rehorst, A. Withagen (8); *Bosch Medicentrum Hospital, Den Bosch* —

H. Dohmen (8); *Martini Hospital, Groningen* — P. Bernink, M. Niemeijer,

J. Posma (8); *Hospital de Tjongerschans, Heerenveen* — S. Oei, J. van Os, G.

Jochemsen (8); *Hospital Medisch Centrum, Leeuwarden* — R. Breedveld,

W. Schenkel, C. de Vries (8); *University Hospital, Maastricht* — C. Kirchhof

(8); *Haven Hospital, Rotterdam* — C. Leenders (7); *de Honte Hospital,*

*Terneuzen* — R. Ciampricotti, R. Taverne, G. Paulussen (6); *Albert Schweitzer*

*Hospital, Dordrecht* — P. Breuls (5); *Ikazia Hospital, Rotterdam* — J. Kerker

(5); *Hospital Refaja, Stadskanaal* — L. Van Wijk (3); *St. Elisabeth Hospital,*

*Tilburg* — W. Pasteuning, N. Holwerda (3); *Albert Schweitzer Hospital,*

*Zwijndrecht* — A. Herweijer (3); *Delfzicht Hospital, Delfzijl* — J. Spanjaard

(3); *University Hospital, Nijmegen* — F. Verheugt (2); *Hospital de Sionsberg,*

*Dokkum* — A. Hagoort-Kok, E. van den Toren (2); *Schieland Hospital,*

*Schiedam* — H. Werner, H. Spierenburg (2); *Policy Advisory Board* — H.

Wellens, K. Lie, N. Van Hemel; *End Point Committee* — J. Van Der Meer,

J. Viersma, M. Van De Linde, A. De Jager; *Steering Committee* — H. Crijns,

I. Van Gelder, H. Bosker, O. Kamp, J. Kingma, J. Tijssen.

**RACE-II**

**Steering committee:** The steering committee includes Isabelle C. Van Gelder (chair), MD, Maarten P. Van den Berg, MD, Harry J.G.M. Crijns, MD, Ype S. Tuininga, MD, A. Marco Alings, MD, Hans A. Bosker, MD, Jan H. Cornel, MD, Otto Kamp, MD, Jan G.P. Tijssen, PhD, Nic J.G.M. Veeger, MSc, Dirk

J. Van Veldhuisen, MD.

**End point adjudication committee:** This committee of experts, masked to the treatment assignments, who will adjudicate all possible end points includes Jan Van der Meer, MD, hematologist (chair, deceased), Johan Brügemann, MD, cardiologist, and Gert-Jan Luijckx, MD, neurologist.

**Advisory board:** The advisory board includes A. John Camm, MD, D. George Wyse, MD, PhD, Albert L. Waldo, MD, John G.F. Cleland, MD.

**Data Safety and Monitoring Board:** The data safety and monitoring board includes Hein J.J. Wellens, MD (chair, deceased), Arthur A.W. Wilde, MD, and Richard N. Hauer, MD.

**Trial and data management:** Trial Coordination Center Groningen

**RACE3**

Participating centers

The Netherlands: G.C.M. Linssen, Hospital Group Twente,

Almelo/Hengelo; G.S. De Ruiter, Onze Lieve Vrouwe

Gasthuis, Amsterdam; H.A. Bosker, Hospital Rijnstate,

Arnhem/Velp; R.H.J. Peters, Ter Gooi Hospital, Blaricum;

M. Alings, Amphia Hospital, Breda; V. Hagens,

Ommelander Hospital Group, location Delfzijl; Y.S.

Tuininga, Deventer Hospital, Deventer; A.H. Liem,

Admiraal de Ruyter Hospital, Goes; R.G. Tieleman,

Martini Hospital, Groningen; I.C. Van Gelder, University

Medical Center Groningen, Groningen; G.J.E. Verdel,

Kennemer Gasthuis, Haarlem; H.J.G.M. Crijns, Maastricht

University Medical Center, Maastricht; G.E. Cramer,

University Medical Center Nijmegen, Nijmegen; A. Van

der Galiën, Ommelander Hospital, Winschoten, J.G.

Meeder, Viecuri Hospital, location Venlo.

United Kingdom: G.Y.H. Lip, Birmingham City

Hospital, Birmingham; J.S. de Bono, University Hospitals

Birmingham, Birmingham; S. Nadar, Good Hope Hospital,

Sutton Coldfield; M.H. Tayebjee, Leeds Teaching Hospitals,

Leeds; C.J. Boos, Poole Hospital, Poole.

**Steering committee**

I.C. Van Gelder1,2 (chair), M. Alings3, H.J.G.M. Crijns4, M.D. Smit1, R. Tukkie5, J. Brügemann1,6, J.R.L.M. Smeets7, F.F. Willems8, H.L. Hillege1,9, J.G. Tijssen10, R.G. Tieleman11, D.J. Van Veldhuisen1, G.Y.H. Lip12.

1Department of Cardiology, Thoraxcenter, University of Groningen, University Medical Center Groningen,

Groningen; 2Interuniversity Cardiology Institute Netherlands, Utrecht; 3Department of Cardiology, Amphia Hospital, Breda; 4Department of Cardiology, Maastricht University Medical Center, Maastricht; 5Department of Cardiology, Kennemer Gasthuis, Haarlem; 6Cardiac Rehabilitation Center, University Medical Center Groningen, Groningen; 7University Medical Center Nijmegen, Nijmegen; 8Hospital Rijnstate, Arnhem/Velp; 9Trial Coordination Center, Department of Epidemiology, University Medical Center Groningen, Groningen; 10Academic Medical Center, Amsterdam; 11Martini Hospital Groningen, Groningen; all the Netherlands; 12Department of Cardiology, City Hospital, Birmingham, United Kingdom.

**Data safety monitoring board**

H.J.J. Wellens1 (chair), A.M. Wilde2, Y.M. Pinto2, J.G.

Tijssen2 (reporter of steering committee).

1Cardiovascular Research Institute, Maastricht; 2Academic

Medical Centre, Amsterdam; all the Netherlands.

**Endpoint adjudication committee**

R.A. Tio (chair) 1, J. van Melle1, G.J. Luijckx2

1Department of Cardiology and 2Neurology, University

Medical Center Groningen, Groningen, the Netherlands.

**Trial management, statistics and data management**

H.L. Hillege1, W.J.M. Mol1, J.G. Tijssen2.

1Trial Coordination Center, University Medical Center

Groningen, Groningen; 2Academic Medical Center,

Amsterdam; all the Netherlands.

**RACE4**

Authors

1. H.J.G.M. Crijns , E.P.J. Wijtvliet, N.A.H.A. Pluymaekers, J.G.L.M. Luermans

2. R.G. Tieleman

3. I.C. Van Gelder, M. Rienstra

4. R. Tukkie

5. R.J. Folkeringa

6. P. Bronzwaer

7. J. Elders

8. A. Elvan

9. A.D.I. Van Asselt

10. S.M.J. Van Kuijk

11. J.G. Tijssen

Institutional affiliations

1. Maastricht University Medical Center (MUMC), Department of Cardiology, Maastricht,

Netherlands (The)

2. Martini Hospital, Department of Cardiology, Groningen, Netherlands (The)

3. University Medical Center Groningen, Department of Cardiology, Groningen, Netherlands (The)

4. Spaarne Hospital, Department of Cardiology, Haarlem, Netherlands (The)

5. Medical Center Leeuwarden, Department of Cardiology, Leeuwarden, Netherlands (The)

6. Zaans Medical Center, Department of Cardiology, Zaandam, Netherlands (The)

7. Canisius Wilhelmina Hospital, Department of Cardiology, Nijmegen, Netherlands (The)

8. Isala Hospital, Department of Cardiology, Zwolle, Netherlands (The)

9. University Medical Center Groningen, Department of Epidemiology, Groningen, Netherlands (The)

10. Maastricht University Medical Centre (MUMC), Department of Clinical Epidemiology and

Medical Technology Assessment, Maastricht, Netherlands (The)

11. Amsterdam University Medical Center, location AMC, Department of Cardiology, Amsterdam,

Netherlands (The)

Members of the RACE4 trial are as follows:

**Writing Committee:** H.J.G.M. Crijns (chair), E.P.J. Wijtvliet, R.G. Tieleman, N.A.H.A. Pluymaekers, I.C. Van Gelder, M. Rienstra, S.M.J. Van Kuijk, J.G.P. Tijssen;

**Steering Committee:** H.J.G.M. Crijns (chair), E.P.J. Wijtvliet, R.G. Tieleman, I.C.

Van Gelder, R. Tukkie, R. Folkeringa, P. Bronzwaer, J. Elders, A. Elvan, M. Rienstra, J.G.P. Tijssen,

A.D.I. Van Asselt, S.M.J. Van Kuijk

**Study statisticians:** Sander van Kuijk and Jan Tijssen

**Investigators** (all in the Netherlands): *Maastricht University Medical Center, Maastricht*— H.J.G.M. Crijns, E.P.J. Wijtvliet, L. Muis-Molin, B. Vorstermans, J.G.L.M. Luermans; *Martini Hospital* — R.G. Tieleman, E.P.J. Wijtvliet, I. Baas, W. de Valk-Bedijn, W. Eijgenraam, I. Eggink, M. Hendriks-van Woerden; *University Medical Center Groningen*, Groningen — I.C. Van Gelder, Y. Blaauw, M. Rienstra, R.R. De With, C.M. Roos, H. Deuling, L. Redelaar, R. Steenbergen, M. Bulten; *Spaarne Hospital*— R. Tukkie, C. Kalkman-Knulst, J. Blonk, E. van Esveld, M. Schmidt-Meijer; *Medical Center Leeuwarden, Leeuwarden*— R. Folkeringa, S. van Arkel-Hoogervorst, M. van der Zee; *Zaans Medical Center, Zaandam*— P. Bronzwaer, E. Lensink, H. Rijks, Y. Scheffer, C. Poel, P. Kruijt, J. Blonk, L. Poerbodipoero; *Canisius Wilhelmina Hospital, Nijmegen*— L. Bouwels, J. Elders, C. Gietemans ; *Isala Hospital, Zwolle* —A. Elvan, J. van Dekken, A. Seppenwoolde, L. van den Brink-Drok, I. Stam, J. Niemeijer-Kamp, J. Visser-Croezen.

**CTCM:** performed the trial monitoring and data management (I. Verkooyen, L. Den Hollander, W. Neyens, M. van Adrichem, L. Knaepen, V. Hellwig, C. Dirksen)

**RACE7-ACWAS**

Authors

1. H.J.G.M. Crijns , N.A.H.A. Pluymaekers, E.A.M.P. Dudink, J.G.L.M. Luermans

2. J.G. Meeder

3. T. Lenderink

4. J. Widdershoven

5. J.J.J. Bucx

6. I.C. Van Gelder, M. Rienstra

7. O. Kamp

8. J.M. Van Opstal

9. M. Alings

10. A. Oomen

11. C.J. Kirchhof

12. V.F. Van Dijk

13. H. Ramanna

14. J.G.P. Tijssen

15. A. Liem

16. L.R. Dekker

17. B.A.B. Essers

Institutional affiliations

1. Maastricht University Medical Center, Cardiology and Cardiovascular Research Institute Maastricht

(CARIM), Maastricht, Netherlands

2. VieCuri Medical Center Noord-Limburg, Cardiology, Venlo, Netherlands

3. Zuyderland Medical Center, Cardiology, Heerlen, Netherlands

4. Elisabeth-TweeSteden Hospital, Cardiology, Tilburg, Netherlands

5. Diakonessen Hospital, Cardiology, Utrecht, Netherlands

6. University Medical Center Groningen, Cardiology, Groningen, Netherlands

7. Amsterdam University Medical Center, location VUmc, Cardiology, Amsterdam, Netherlands

8. Medical Spectrum Twente, Cardiology, Enschede, Netherlands

9. Amphia Hospital, Cardiology, Breda, Netherlands

10. Antonius Hospital, Cardiology, Sneek, Netherlands

11. Alrijne Hospital, Cardiology, Leiderdorp, Netherlands

12. St. Antonius Hospital, Cardiology, Nieuwegein, Netherlands

13. Haga Teaching hospital, Cardiology, The Hague, Netherlands

14. Amsterdam University Medical Center, location AMC, Cardiology, Amsterdam, Netherlands

15. St. Franciscus Gasthuis, Cardiology, Rotterdam, Netherlands

16. Catharina Hospital, Cardiology, Eindhoven, Netherlands

17. Maastricht University Medical Center, Clinical Epidemiology and Medical Technology

Assessment, Maastricht, Netherlands

Members of the RACE 7 ACWAS trial are as follows:

**Writing Committee:** H.J.G.M. Crijns (chair), N.A.H.A. Pluymaekers, E.A.M.P. Dudink, J.G.L.M. Luermans, I.C. Van Gelder, M. Rienstra, J.G. Meeder, J.G.P. Tijssen;

**Steering Committee:** H.J.G.M. Crijns (chair), E.A.M.P. Dudink, N.A.H.A., Pluymaekers, J.G.L.M. Luermans, I.C. Van Gelder, A. Liem, C.J. Kirchhof, V.F. Van Dijk, H. Ramanna, J.J.J. Bucx, J.G. Meeder, J. Widdershoven, J.M. Van Opstal, T. Lenderink, L.R. Dekker, M. Alings, O. Kamp, M. Rienstra, A. Oomen, J.G.P. Tijssen, B.A.B. Essers;

**Investigators** (all in the Netherlands): *Maastricht University Medical Center, Maastricht* — H.J.G.M. Crijns, E.A.M.P. Dudink, N.A.H.A. Pluymaekers, J.G.L.M. Luermans, B. Weijs, W. Holvoet; *VieCuri Medical Center, Venlo* — J.G. Meeder, A. Duygun, F. Körver, I.A. Joosen, T. Lankveld, R. Janssen, E.M. Poels, J. Peute, E. Woelders; *Zuyderland Medical Center, Heerlen* — T. Lenderink, T. J. Ansink, R. Pisters, M. Mafi Rad; *Elisabeth-TweeSteden hospital, Tilburg* — J. Widdershoven, Z. Gocmen, M. Scheurwater, J. Verhagen, W. Fitski; *Diakonessen Hospital, Utrecht* — J.J.J. Bucx; *University Medical Center Groningen, Groningen* — I. C. Van Gelder, M. Rienstra, A.H. Hobbelt, R.R. De With, L.B.O. Nguyen; *Amsterdam University Medical Center, location VUmc, Amsterdam* — O. Kamp, M. Ek, B. Koopman, E.J. Verduyn; *Medical Spectrum Twente, Enschede* — J.M. Van Opstal, L. Oldenhof, M. Bader, H. Verheij; *Amphia Hospital, Breda* — M. Alings, J. Huijbregtse, I. Hunze; *Antonius Hospital,Sneek* — A. Oomen, C. Terwisscha van Scheltinga; *Alrijne Hospital, Leiderdorp* — C. J. Kirchhof, L. Kulker; *St. Antonius hospital, Nieuwegein* — V.F. Van Dijk, B. Kara; *Haga Teaching Hospital, The Hague* — H. Ramanna; H. de Lange- van Bruggen; E. Karijodikoro; *St. Franciscus Gasthuis, Rotterdam* — A. Liem, I. Kort; *Catharina Hospital, Eindhoven* — L.R. Dekker, L.M. Rademakers.

**Statistical analysis committee:** J.P.G. Tijssen (Chair), N.A.H.A. Pluymaekers M. Rienstra, H.J.G.M. Crijns, E.A.M.P. Dudink, I.C. van Gelder

**Trial and data management:** Clinical Trial Center Maastricht, L. van Kan, M. van Adrichem, I. Verkooijen, L. den Hollander, W. Neyens, K. Diepvens, C Dircksen
